# Effects of Oxaliplatin on Facial Sensitivity to Cool Temperatures and TRPM8 Expressing Trigeminal Ganglion Neurons in Mice

**DOI:** 10.3389/fpain.2022.868547

**Published:** 2022-05-11

**Authors:** Robert M. Caudle, John K. Neubert

**Affiliations:** ^1^Department of Oral and Maxillofacial Surgery, University of Florida, Gainesville, FL, United States; ^2^Department of Orthodontics, University of Florida, Gainesville, FL, United States

**Keywords:** oxaliplatin (43805), paclitaxel, chemotherapy, trigeminal ganglia, face, patch clamp, orofacial pain, TRPM8

## Abstract

The chemotherapeutic agent oxaliplatin is commonly used to treat colorectal cancer. Although effective as a chemotherapeutic, it frequently produces painful peripheral neuropathies. These neuropathies can be divided into an acute sensitivity to cool temperatures in the mouth and face, and chronic neuropathic pain in the limbs and possible numbness. The chronic neuropathy also includes sensitivity to cool temperatures. Neurons that detect cool temperatures are reported to utilize Transient Receptor Potential Cation Channel, Subfamily M, Member 8 (TRPM8). Therefore, we investigated the effects of oxaliplatin on facial nociception to cool temperatures (18°C) in mice and on TRPM8 expressing trigeminal ganglion (TRG) neurons. Paclitaxel, a chemotherapeutic that is used to treat breast cancer, was included for comparison because it produces neuropathies, but acute cool temperature sensitivity in the oral cavity or face is not typically reported. Behavioral testing of facial sensitivity to 18°C indicated no hypersensitivity either acutely or chronically following either chemotherapeutic agent. However, whole cell voltage clamp experiments in TRPM8 expressing TRG neurons indicated that both oxaliplatin and paclitaxel increased Hyperpolarization-Activated Cyclic Nucleotide-Gated channel (HCN), voltage gated sodium channel (Na_v_), and menthol evoked TRPM8 currents. Voltage gated potassium channel (K_v_) currents were not altered. Histological examination of TRPM8 fibers in the skin of the whisker pads demonstrated that the TRPM8 expressing axons and possible Merkel cell-neurite complexes were damaged by oxaliplatin. These findings indicate that oxaliplatin induces a rapid degeneration of TRG neuron axons that express TRPM8, which prevents evoked activation of the sensitized neurons and likely leads to reduced sensitivity to touch and cool temperatures. The changes in HCN, Na_v_, and TRPM8 currents suggest that spontaneous firing of action potentials may be increased in the deafferented neurons within the ganglion, possibly producing spontaneously induced cooling or nociceptive sensations.

## Introduction

According to the Center for Disease Control ~650,000 people receive chemotherapy for cancer each year in the United States. A significant number of these patients will develop painful peripheral neuropathies because of their treatment. The pain from the neuropathies may lead to the curtailment of therapy, which may have a negative impact on the patient's prognosis. Most of the pain and other symptoms associated with the neuropathies are associated with the limbs. Pain in orofacial regions is typically only reported following platinum-based antineoplastic drugs like oxaliplatin (1–3). Oxaliplatin frequently produces an acute sensitivity to cool or cold temperatures in the face or mouth. This usually resolves quickly and simply requires the patient to avoid cold food or drinks for a few days. The acute symptoms are often followed by a more long-term neuropathy in the limbs (1–11). The acute trigeminal mediated orofacial sensitivity to cool temperatures suggests that temperature sensitive trigeminal ganglion neurons may be particularly vulnerable to oxaliplatin toxicity. We have previously examined Transient Receptor Potential cation channel subfamily M (melastatin) member 8 (TRPM8) in trigeminal ganglion (TRG) neurons and the role these neurons play in facial temperature sensation (12). TRPM8 is a temperature sensing ion channel that is activated by temperatures of <22°C (13). Although TRPM8 did not participate in the response of the TRG neurons to cool temperatures in our previous study the TRPM8 expressing neurons did become more excitable when the neurons were cooled. Previous studies have demonstrated that treating rodents with oxaliplatin increases transcription of TRPM8 in TRG neurons (5, 8). The increased transcription was maintained for several weeks indicating that the TRPM8 expressing TRG neurons may also contribute to the overall neuropathy. Furthermore, Descoeur et al. demonstrated that TRPM8 knockout mice displayed significantly less cool allodynia following oxaliplatin treatment than wildtype mice (8). Thus, we hypothesized that TRPM8 expressing TRG neurons would mediate cool temperature allodynia in the face of mice following oxaliplatin treatment. This project examined the effect of oxaliplatin on cool and warm sensitivity in the face of mice and the impact oxaliplatin treatment had on ion channel function in TRPM8 expressing TRG neurons.

## Methods

### Mice

Male and female hairless SKH1 mice (20–35 g, Charles Rivers, Wilmington, MA) were utilized for behavioral experiments. The hairless phenotype allows direct contact of the animal's skin with the thermal probes on the testing apparatus, thus eliminating the insulating effect of the fur. Additionally, shaving of furred mice leads to skin irritation that may induce hypersensitivity in the skin (14).

TRPM8^tm1Apat^/J knockout mice (Jackson Labs, Bar Harbor, ME) were crossed in house with C57BL/6 mice (Charles Rivers, Wilmington, MA) to produce heterozygotes (TRPM8^EGFP−/+^). The TRPM8^tm1Apat^/J knockout mice express enhanced green fluorescent protein (eGFP) in place of TRPM8. Male and female heterozygotes (TRPM8^EGFP−/+^) were utilized for electrophysiology and immunohistochemistry of TRPM8 expressing neurons.

The animals were housed with a 12-h light–dark cycle, and food and water were available *ad libitum*. Experiments were conducted between 09.00 and 18.00 h at a room temperature of ~22°C. All experiments were approved by the University of Florida Institutional Animal Care and Use Committee and were performed in compliance with the National Institutes of Health guidelines.

### Behavior

**Figure 1:**
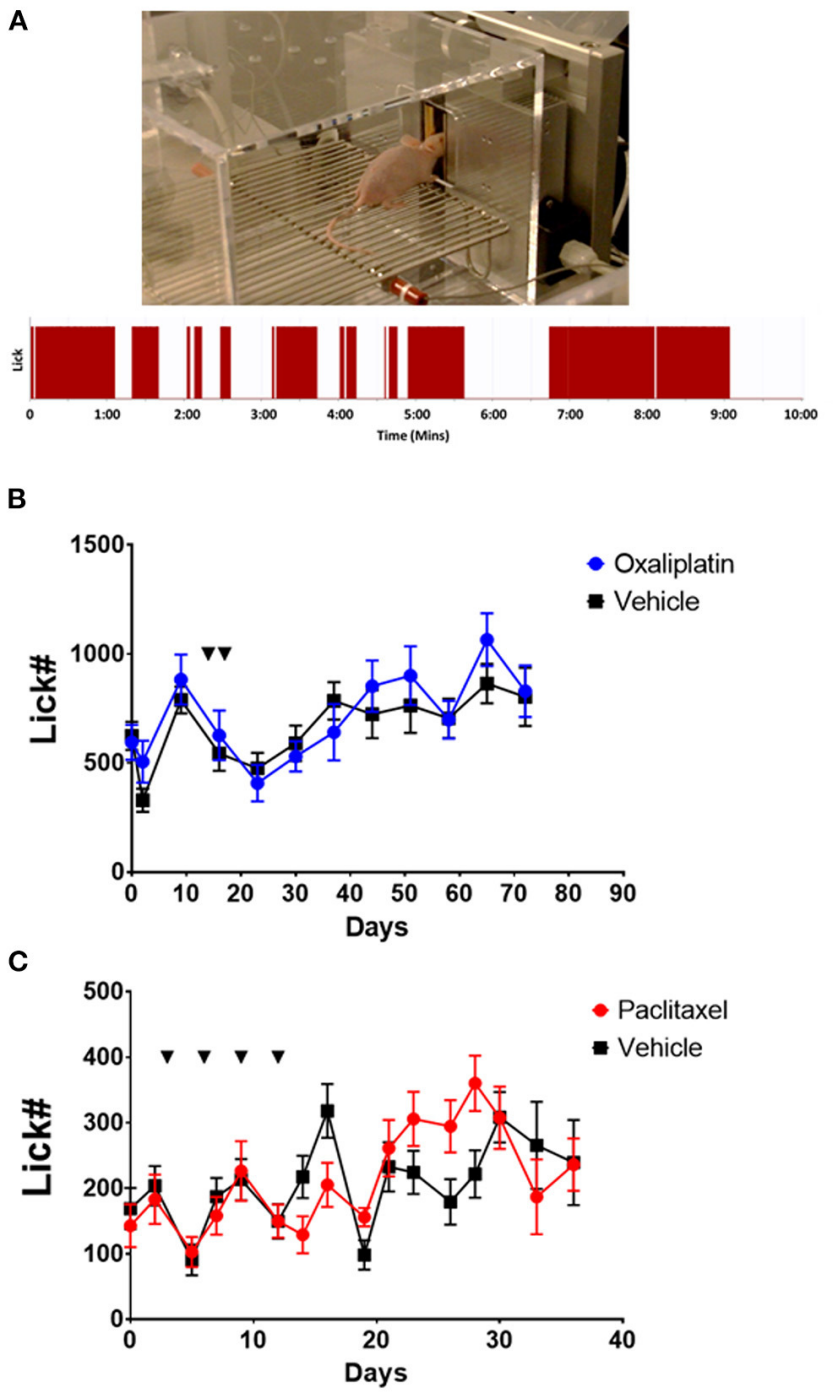
Nociceptive responses to 18°C of SKH1 mice following oxaliplatin or paclitaxel treatment. **(A)** Example of a hairless SKH1 mouse performing in the OPAD assay. The trace below the picture is a representative example of the raw licking data collected by AnyMaze in the OPAD. **(B)** Oxaliplatin (10 mg/kg, ip. injections indicated by the arrows) did not influence the response of the mice to 18°C in the OPAD assay [2-Way Repeated Measures ANOVA *F*_(1, 40)_ = 0.9409, *P* = 0.3379, *N* = 10 oxaliplatin, *N* = 32 vehicle]. **(C)** Paclitaxel (26 mg/kg, ip. injections indicated by the arrows) did not influence the response of the mice to 18°C in the OPAD assay [2-Way Repeated Measures ANOVA *F*_(1, 38)_ = 0.02492, *P* = 0.8754, *N* = 20 paclitaxel, *N* = 20 vehicle].

Hairless SKH1 mice of both sexes (Charles Rivers, Wilmington, MA, 20–35 g) were tested in Orofacial Pain Assessment Devices (OPAD, Stoelting Co., Wood Dale, IL). OPADs utilize a reward/conflict paradigm that requires the mice to place their faces onto temperature programmable Peltier bars to obtain a reward solution (14–27) (Figure 1A). Licking on the reward bottle is captured by the animal completing a circuit when they contact the reward bottle with their tongue (see example data in Figure 1A). The total number of licks on the bottle during the testing session was used as the dependent measure. The reward solution consisted of sweetened condensed milk diluted 1:2 with water. The animals' food was removed from their home cages 12–15 h prior to testing, but they received water *ad libitum* throughout the fasting period.

For experiments the mice were trained over a 2-week period at three sessions per week with the Peltier bars set at room temperature. Sessions were 10 min in length. The OPADs were then programed to test the animals at 18° or 42°C during 10-min sessions while recording licks on the reward bottle. Baseline responses at 18° and 42°C were collected prior to administering oxaliplatin or paclitaxel. The animals were tested three times per week for up to 80 days.

### Chemotherapeutic Treatment

The oxaliplatin treated mice were given two injections of oxaliplatin (10 mg/kg, ip.) (Sigma Aldrich, St. Louis MO) dissolved in phosphate buffered saline (PBS). The injections were separated by 3 days. Vehicle control animals received two injections of an equal volume of PBS. The paclitaxel treated mice received four injections of paclitaxel (26 mg/kg, ip.) (Sigma Aldrich, St. Louis MO) spaced 3 days apart. The paclitaxel was dissolved in DMSO. Vehicle treated animals received four injections of an equal volume of DMSO.

### Electrophysiology

TRPM8^EGFP−/+^ mice were euthanized by isoflurane inhalation (5% in O_2_) and the trigeminal ganglia were removed. The ganglia were then incubated for 2 h at 37° in Tyrode's buffer (mM: 140 NaCl, 4 KCl, 2 MgCl_2_, 2 CaCl_2_, 10 glucose, and 10 HEPES, adjusted to pH 7.4 with NaOH) containing collagenase (Sigma Aldrich, St. Louis, MO) (2 mg/ml). The ganglia were triturated with a plastic pipette, pelleted by centrifugation (100 X g), resuspended with fresh Tyrode's buffer, and plated onto 30 mm polystyrene plates. The cells were allowed to adhere to the plates for 1 h at room temperature prior to initiating the experiments (28–30). During the experiments the cells were superfused with Tyrode's buffer at 5 mls per minute at room temperature (~22°C). The bath's volume was maintained at ~1 ml. The TRPM8 expressing neurons were identified by the expression of eGFP using an inverted microscope equipped with fluorescence optics (Olympus IX70). EGFP labeled cells were whole cell patch clamped with 1.5 mm glass electrodes filled with electrode buffer consisting of (mM: 140 KCl, 1 CaCl_2_, 10 EGTA, 10 HEPES, 2 MgCl_2_). The pH was adjusted to 7.4 with KOH. The electrodes were pulled to resistances of 2–4 MΩ with a P-87 Flaming/Brown microelectrode puller (Sutter Instruments, Navato, CA). Data were collected using an Axopatch 200B amplifier, a Digidata 1200 analog to digital converter and PClamp8 software. Following establishment of the whole cell voltage clamp configuration the series resistance was compensated by 50–60% and cell capacitance was compensated using the settings on the amplifier.

**Figure 2:**
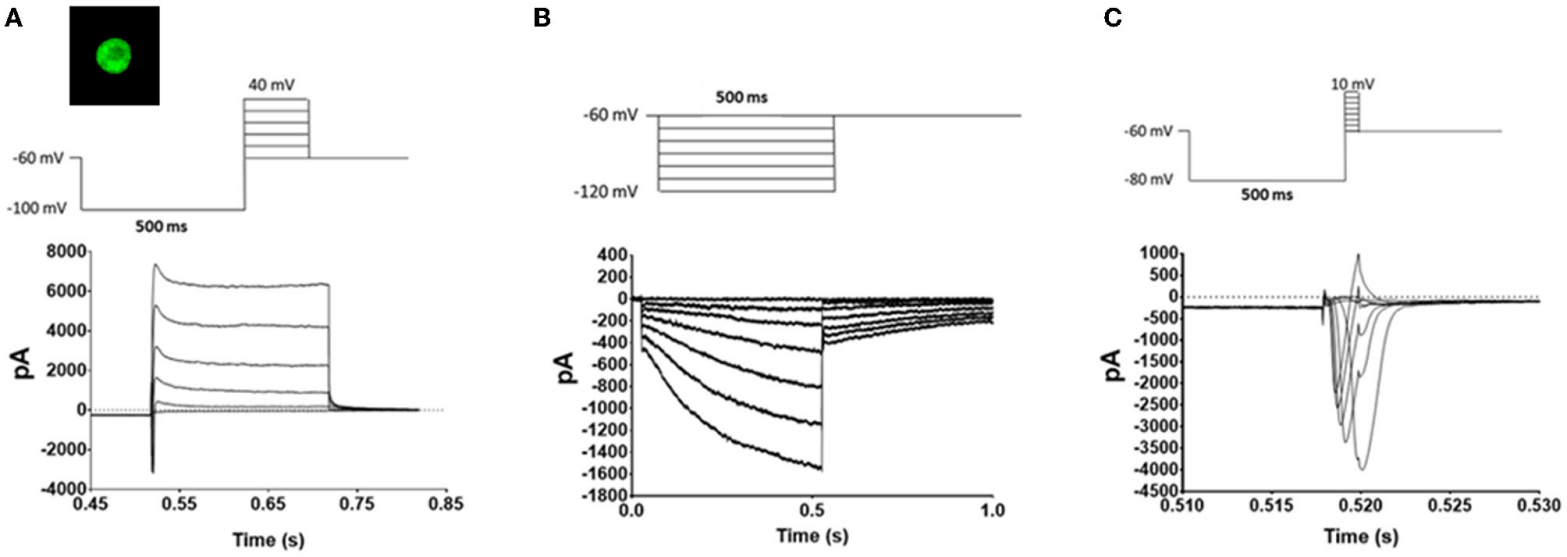
Voltage protocols. TRPM8 expressing TRG neurons were identified by eGFP fluorescence (Image) and whole cell voltage clamped. Once a giga seal was obtained and the patch was removed the neurons underwent a series of voltage protocols to evaluate the effects of oxaliplatin and paclitaxel on K_v_ mediated currents **(A)**, HCN mediated currents **(B)**, and Na_v_ mediated currents **(C)**. The top traces are the voltage protocols, and the bottom traces are representative currents generated by the protocols in TRPM8 expressing TRG neurons.

Voltage protocols for voltage gated potassium channels (K_v_), hyperpolarization-activated cyclic nucleotide-gated channels (HCN), and voltage gated sodium channels (Na_v_) were as previously described (28, 30–32). Briefly, to activate K_v_ channels the cells were hyperpolarized to −100 mV for 500 ms and then stepped from −60 to 40 mV in 20 mV increments for 200 ms (Figure 2A). The HCN channels were activated by hyperpolarizing the cell membrane stepwise in 10 mV increments from −60 to −120 mV for 500 ms (Figure 2B). Na_v_ currents were activated by hyperpolarizing the cell membrane to −100 mV for 500 ms and then stepping the potential in 10 mV increments from −60 mV to 10 mV for 2 ms (Figure 2C).

To activate TRPM8 currents menthol (100 μM) was added to the Tyrode's buffer while holding the cells at −60 mV.

To calculate ion channel conductance the following equation was used:


(1) $$\begin{array}{r} g=\frac{I}{\left(V-V_{r}\right)}\;\left(1\right) \end{array}$$

where g is the conductance, I is the measured current, V is the command voltage, and V_r_ is the reversal potential of the current. To evaluate the probability of channel opening at a given membrane potential the conductance was normalized. Normalization was performed by taking the ratio of the measured conductance to the maximum conductance.

### Modeling Membrane Properties of TRPM8 Expressing TRG Neurons

The stability of the membrane potential in neurons with pacemaker like properties is determined by the interaction of HCN channels and K_v_ channels (33). Therefore, the behavior of the TRPM8 neurons was evaluated by modeling the relationship between HCN and K_v_ channels using the modified Nernst equation:


(2) $$\begin{array}{r} V_{m\left(t\right)}=\frac{RT}{zF}\cdot ln\frac{P_{h\left(t-n\right)}{\left[Na^{+}\right]}_{out}+P_{k\left(t-1\right)\;}{\left[K^{+}\right]}_{out}}{P_{h\left(t-n\right)}{\left[Na^{+}\right]}_{in}+P_{k\left(t-1\right)}{\left[K^{+}\right]}_{in}} \end{array}$$

Where V_m(t)_ is the membrane potential at time t, R is the gas constant, T is temperature in Kelvin, F is the Faraday constant, and z is the valence of the ions. The relative difference in the time constants (τ) for HCN and K_v_ channels was compensated for by using the variable n. At a temperature of 22°C the value of RT/zF is 24.89. P_h_ and P_k_ are the relative permeability of HCN channels and K_v_ channels, respectively. The membrane potentials at time t-n and t-1 were used to estimate ion permeability using lookup tables generated from non-linear curve fitting of conductance produced from the ion channels' current/voltage relationships (12). These permeabilities were used to calculate the membrane voltage at time t to generate a time series.

### Immunohistochemistry

TRPM8^EGFP−/+^ mice (2 oxaliplatin treated, 2 vehicle treated) were euthanized by isoflurane inhalation (5% in O_2_) and perfused intracardially with PBS (pH 7.4) and subsequently 4% paraformaldehyde 6 weeks after oxaliplatin treatment. The skin from the whisker pads was shaved and removed. The tissue was fixed for 15 h in 4% paraformaldehyde, and then transferred to 30% sucrose in PBS for 24–30 h. The tissues were frozen and transverse sections cut at 40 μm. Five sections from each mouse were collected. The free-floating sections were incubated in PBS, 0.3% Triton X-100 and 5% normal goat serum (NGS–T) for 1 h before incubation in primary anti-green fluorescent protein antibody (Invitrogen, Waltham, MA) at a dilution of 1:50,000 in NGS-T for 18 h at room temperature. Preparations were then rinsed in NGS-T 3 times for 10 min each. The secondary antibody, Alexa Fluor 594 goat anti-rabbit IgG (Invitrogen, Waltham, MA), was diluted to 1:500 with NGS-T and the preparations incubated at room temperature for 1 h. The sections were then rinsed 3 times for 10 min each, mounted, cover slipped, and imaged. Images were taken of all fluorescent labeled basket like structures in the tissues. The images were then processed in ImageJ by outlining the labeled basket like structures and measuring the area of the structures.

### Statistics

Data on the OPADs was collected using AnyMaze (Stoelting Co., Wood Dale, IL). Data collected with PClamp8 were analyzed with Clampfit (Axon Instruments). Immunohistological data were analyzed with ImageJ. PRISM6 (GraphPad Software Inc., La Jolla, CA) was used for statistical analysis. The data were subjected to *T*-Tests, One-Way ANOVAs, Two-Way Repeated Measures ANOVAs, or non-linear regressions as appropriate. Dunnett's test or Bonferroni's multiple comparisons test were used for *post-hoc* analysis. Alpha was set to 0.05 for all experiments. All data are expressed as means ± SEM.

## Results

### Behavioral Effects of Oxaliplatin and Paclitaxel in the OPAD Assay

A common side effect of oxaliplatin chemotherapy is an acute hypersensitivity to cool temperatures, particularly in orofacial regions. The acute sensitivity usually resolves, but then a more chronic painful peripheral neuropathy may develop, which can become severe enough to necessitate curtailing therapy (1–5, 8, 11, 34). We evaluated the effects of oxaliplatin on cool temperature hypersensitivity in the face of hairless SKH1 mice using an OPAD assay (Figure 1A). Since paclitaxel is not associated with acute cool sensitivity but does produce a chronic painful peripheral neuropathy, we included paclitaxel as a comparator.

Treating SKH1 hairless mice with oxaliplatin did not produce hypersensitivity to an 18°C stimulus in the OPAD assay either acutely, tested within 5 h of the first treatment, or chronically [Figure 1B, 2-Way Repeated Measures ANOVA *F*_(1, 40)_ = 0.9409, *P* = 0.3379, *N* = 10 oxaliplatin, *N* = 32 vehicle]. Male and female mice did not differ in their responses so the data for male and female mice were combined [2-Way Repeated Measures ANOVA *F*_(1, 18)_ = 3.490e-005, *P* = 0.9954]. Similarly, paclitaxel did not produce hypersensitivity in the assay at 18°C [Figure 1C, 2-Way Repeated Measures ANOVA *F*_(1, 38)_ = 0.02492, *P* = 0.8754, *N* = 20 paclitaxel, *N* = 20 vehicle]. In contrast, paclitaxel treated mice demonstrated persistent hypersensitivity in the OPAD to 42°C [2-Way Repeated Measures ANOVA *F*_(1, 34)_ = 10.45, *P* = 0.0027, *N* = 16 paclitaxel, *N* = 20 vehicle], while oxaliplatin did not produce hypersensitivity to 42°C [2-Way Repeated Measures ANOVA *F*_(1, 44)_ = 0.1215, *P* = 0.7290, *N* = 10 oxaliplatin, *N* = 36 vehicle] (Supplementary Figure 1).

### Electrophysiological Effects of Oxaliplatin and Paclitaxel on TRPM8 Expressing TRG Neurons

The lack of effect of both oxaliplatin and paclitaxel on nociception in the face of the mice at 18°C was unexpected given the literature on their effects in the limbs (5, 8–10, 34–44). Dorsal root ganglia (DRG) neurons that express TRPM8 are sensitized by oxaliplatin and believed to mediate the acute cool temperature hypersensitivity. The DRG TRPM8 neurons are also involved in oxaliplatin's chronic painful peripheral neuropathy (5, 8, 9). To determine if the chemotherapeutic agents also altered the function of TRPM8 expressing TRG neurons we treated TRPM8^EGFP−/+^ mice with oxaliplatin or paclitaxel as described in the methods and then harvested TRG neurons 6–10 weeks following the last injection of the chemotherapeutic agent. The TRPM8^EGFP−/+^ mice express eGFP in TRPM8 expressing neurons. The TRG neurons were dissociated, plated, and then TRPM8 eGFP expressing neurons were identified by their fluorescence (image in Figure 2A). The labeled neurons were whole cell voltage clamped. The standardized voltage protocols for K_v_ currents (Figure 2A), HCN currents (Figure 2B), and Na_v_ currents (Figure 2C) (12, 28) as described in the methods were used to characterize the long-term effects of the agents on the TRPM8 expressing TRG neurons.

After the whole cell voltage clamp was established the holding currents at −60mV were determined for the TRPM8 expressing neurons. The holding currents did not differ when comparing vehicle treated animals to either oxaliplatin or paclitaxel treated animals with currents of −44.95 ± 11.74 pA, −54.39 ± 12.20 pA, and −37.29 ± 12.87 pA, respectively [ANOVA *F*_(2, 153)_ = 0.2519, *P* = 0.7777, *N* = 91 neurons from 14 vehicle treated mice, *N* = 40 neurons from 9 oxaliplatin treated mice, and *N* = 25 neurons from 5 paclitaxel treated mice].

**Figure 3:**
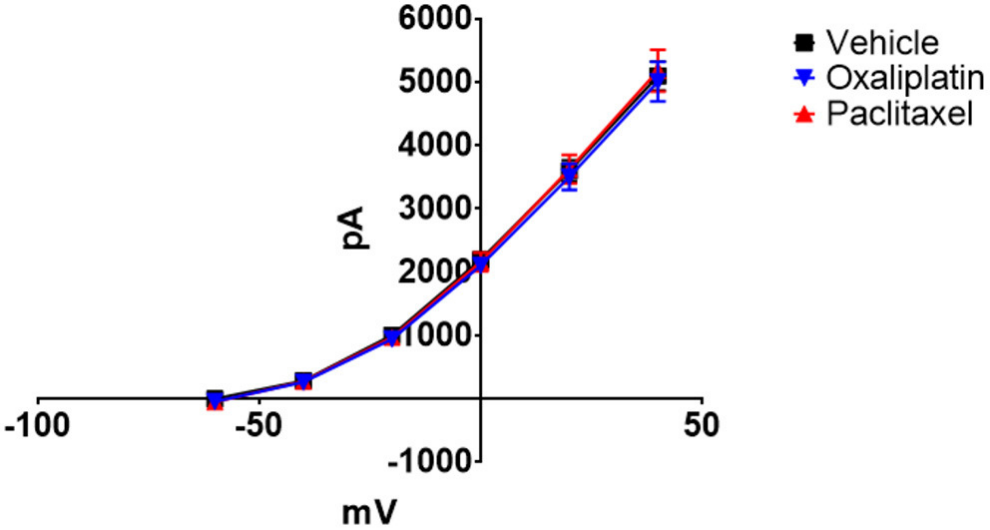
Effect of oxaliplatin and paclitaxel on K_v_ currents in isolated TRPM8 expressing TRG neurons. TRPM8^EGFP−/+^ mice were treated with oxaliplatin, paclitaxel, or PBS ip. as described in the methods. The mice were euthanized 6–10 weeks following the treatments and TRG were isolated, dissociated, plated, and voltage clamped. Neither oxaliplatin nor paclitaxel influenced the K_v_ currents [2-Way Repeated Measures ANOVA *F*_(2, 180)_ = 0.06814, *P* = 0.9342, *N* = 120 neurons from 14 vehicle treated mice, *N* = 34 neurons from 9 oxaliplatin treated mice, *N* = 29 neurons from 5 paclitaxel treated mice].

**Figure 4:**
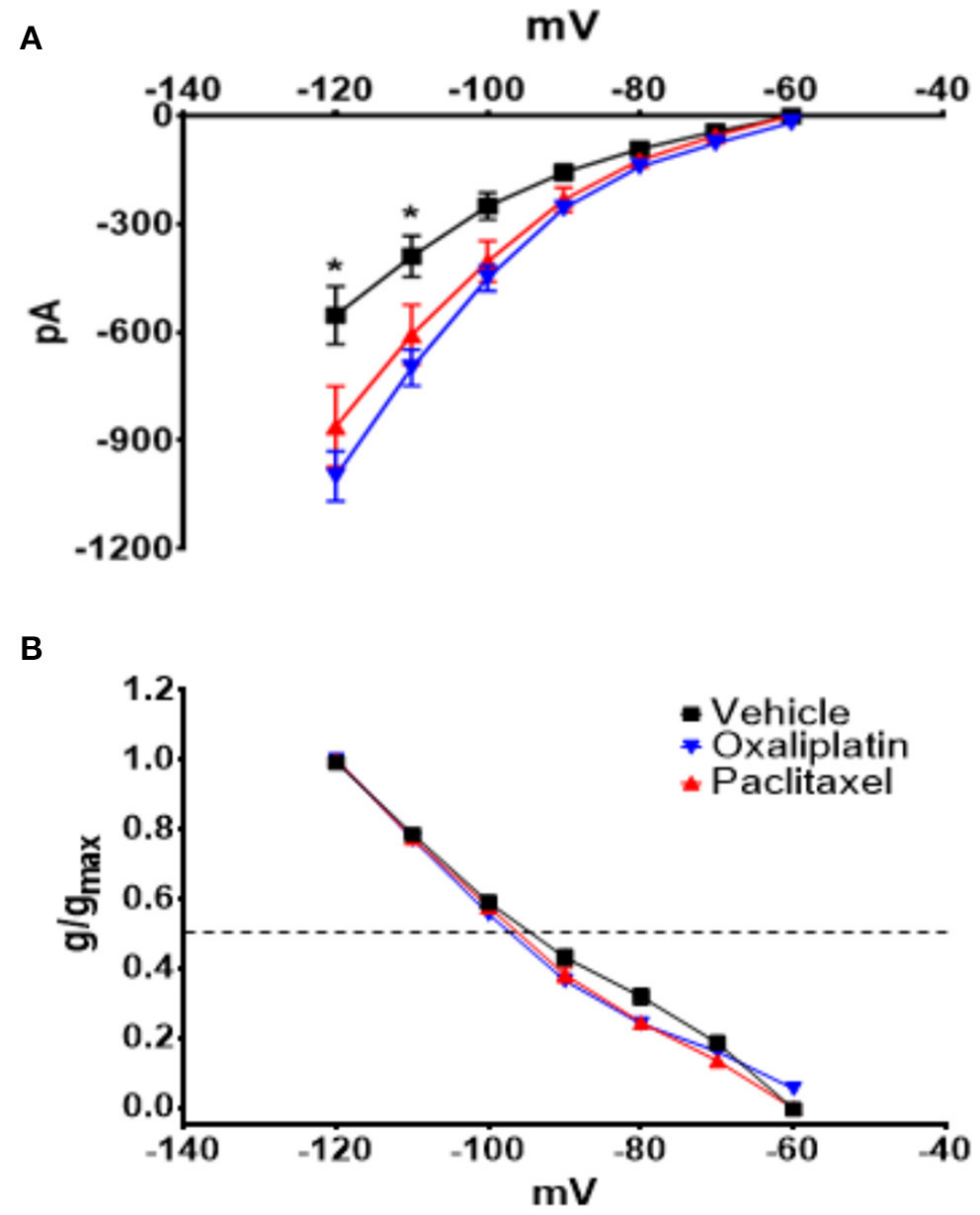
Effect of oxaliplatin and paclitaxel on HCN currents in isolated TRPM8 expressing TRG neurons. Mice were treated with the chemotherapeutic agents and the TRPM8 expressing TRG neurons were voltage clamped as previously described. **(A)** Oxaliplatin and paclitaxel increased the amplitude of HCN currents [2-Way Repeated Measures ANOVA *F*_(2, 756)_ = 30.94, *P* < 0.0001, *N* = 51 neurons from 14 vehicle treated mice, *N* = 39 neurons from 10 oxaliplatin treated mice, *N* = 21 neurons from 5 paclitaxel treated mice]. **(B)** Normalized conductance for HCN currents [2-Way Repeated Measures ANOVA *F*_(2, 136)_ = 2.227, *P* = 0.1118, *N* = 27 neurons from 14 vehicle treated mice, *N* = 32 neurons from 9 oxaliplatin treated mice, *N* = 20 neurons from 5 paclitaxel treated mice]. The horizontal dotted line represents 50% activation of the currents.

Figure 3 demonstrates that both oxaliplatin and paclitaxel had no effect on K_v_ currents [2-Way Repeated Measures ANOVA *F*_(2, 180)_ = 0.06814, *P* = 0.9342, *N* = 120 neurons from 14 vehicle treated mice, *N* = 34 neurons from 9 oxaliplatin treated mice, *N* = 29 neurons from 5 paclitaxel treated mice]. HCN currents, on the other hand, were significantly increased by oxaliplatin and paclitaxel treatment (Figure 4A) [2-Way Repeated Measures ANOVA *F*_(2, 756)_ = 30.94, *P* < 0.0001, *N* = 51 neurons from 14 vehicle treated mice, *N* = 39 neurons from 10 oxaliplatin treated mice, *N* = 21 neurons from 5 paclitaxel treated mice]. Normalizing the HCN conductance demonstrated that the activation threshold for the currents was not altered by the agents (Figure 4B) [2-Way Repeated Measures ANOVA *F*_(2, 136)_ = 2.227, *P* = 0.1118].

**Figure 5:**
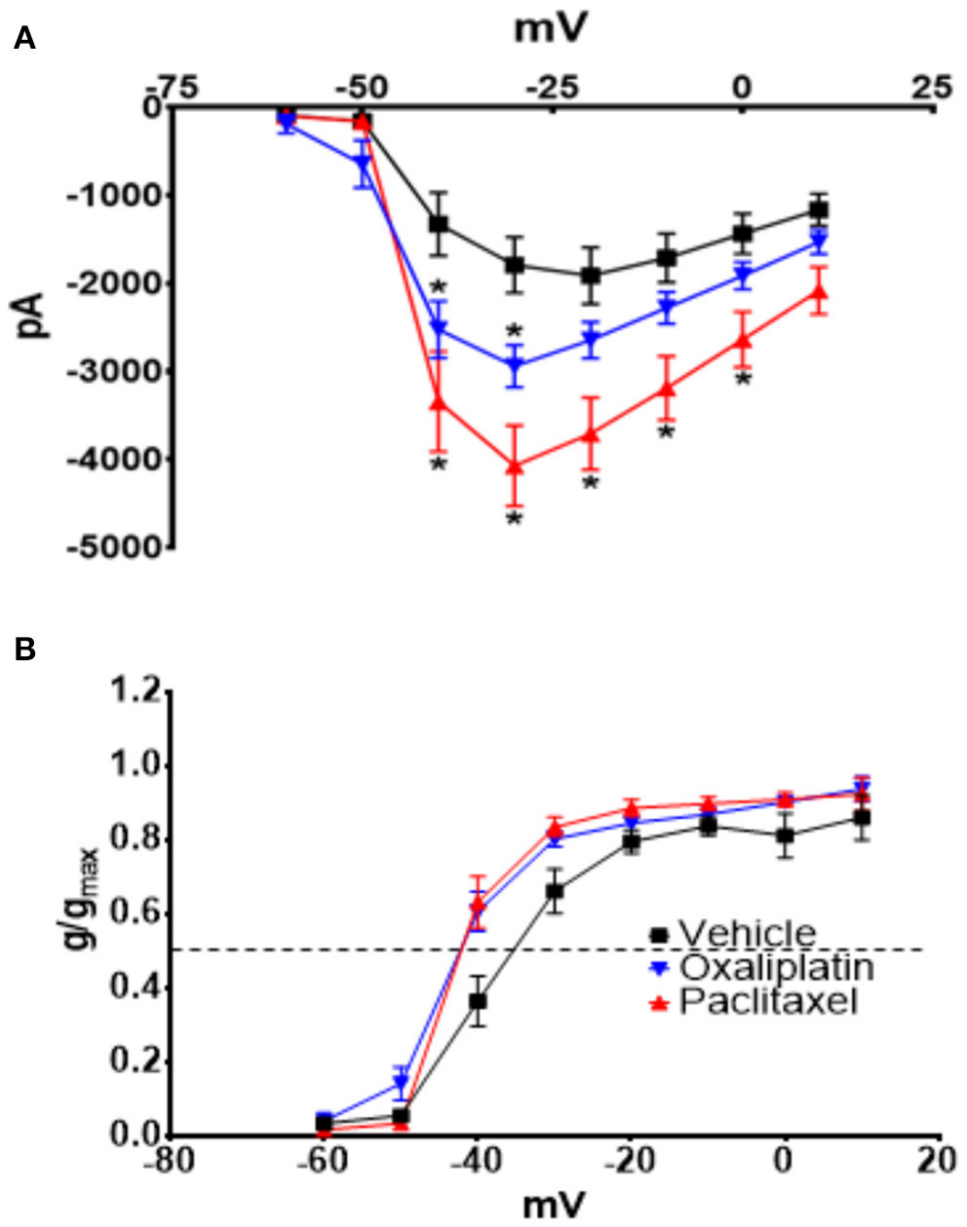
Effect of oxaliplatin and paclitaxel on Na_v_ currents in isolated TRPM8 expressing TRG neurons. Mice were treated with the chemotherapeutic agents and the TRPM8 expressing TRG neurons were voltage clamped as previously described. **(A)** Oxaliplatin and paclitaxel increased peak Na_v_ currents [2-Way Repeated Measures ANOVA *F*_(2, 616)_ = 38.49, *P* < 0.0001]. **(B)** The normalized conductance for Na_v_ currents demonstrates that oxaliplatin and paclitaxel shift the activation potential to more negative voltages [2-Way Repeated Measures ANOVA *F*_(2, 75)_ = 9.801, *P* = 0.0002]. Asterisks indicate *P* < 0.05 using Bonferroni's multiple comparisons test when compared to vehicle at that time point.

Na_v_ currents were also significantly increased by both agents (Figure 5A) [2-Way Repeated Measures ANOVA *F*_(2, 616)_ = 38.49, *P* < 0.0001, *N* = 27 neurons from 14 vehicle treated mice, *N* = 32 neurons from 9 oxaliplatin treated mice, *N* = 20 neurons from 5 paclitaxel treated mice]. The normalized Na_v_ conductance, however, demonstrated that the activation threshold shifted to more negative potentials (Figure 5B) [2-Way Repeated Measures ANOVA *F*_(2, 75)_ = 9.801, *P* = 0.0002]. The 50% activation potential for Na_v_ currents in TRG neurons isolated from vehicle, oxaliplatin, and paclitaxel treated mice were −38.07 ± 1.46 mV, −42.96 ± 0.87 mV, and −42.15 ± 0.87 mV, respectively.

**Figure 6:**
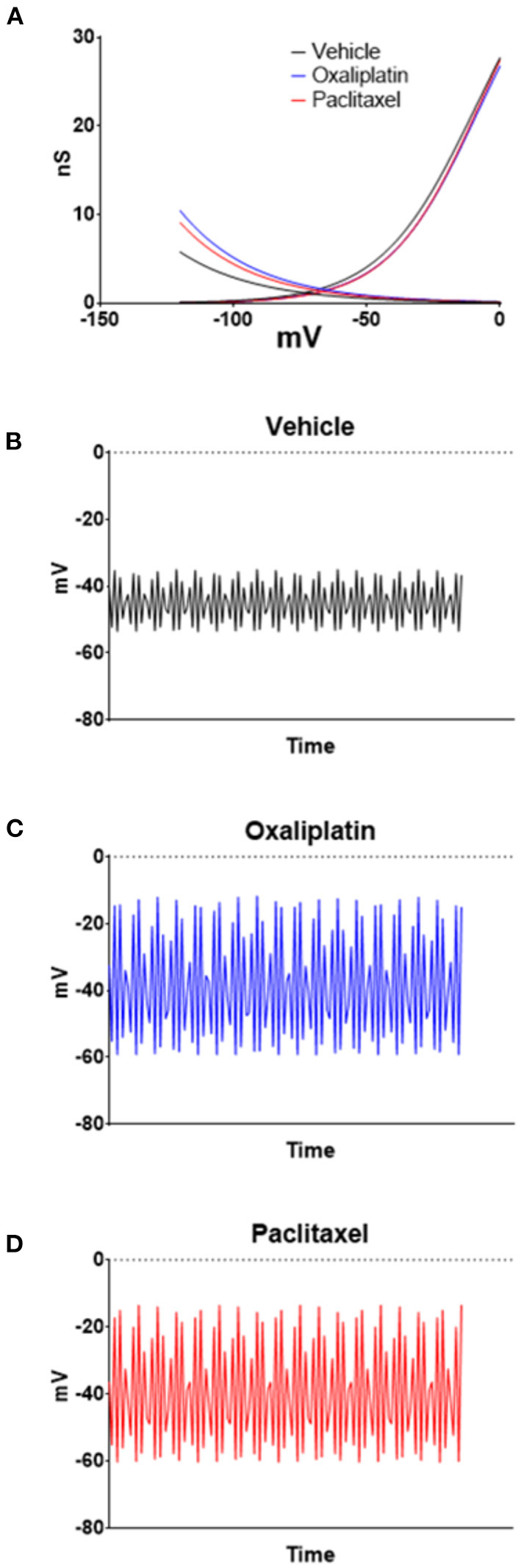
Modeling the effects of oxaliplatin and paclitaxel on TRMP8 expressing TRG neurons' membrane potential. Non-linear regressions were performed on the HCN and K_v_ conductances to generate lookup tables **(A)**. The lookup tables were then used to calculate the neuron membrane voltage time series for vehicle **(B)**, oxaliplatin **(C)**, and paclitaxel **(D)** treated TRPM8^EGFP−/+^ mice using Equation (2). For the examples *n* = 5 was used in equation 2; however, similar results are obtained with values of *n* from 3 to 20.

HCN currents are responsible for pacemaker activity in neurons and cardiac cells (45–47), which suggests that the increase in HCN currents produced by the chemotherapeutic agents could decrease membrane stability and induce spontaneous firing. In pacemaker types of cells, the balance between HCN and K_v_ channels determines the relative firing rate of the cells (33). Therefore, to examine the relationship of the HCN currents to membrane voltage changes the HCN and K_v_ conductance/voltage relationships were curve fitted by non-linear regression. The curves were then used as lookup tables to model membrane voltages using equation 2 as previously described (12). Equation 2 estimates the interaction between HCN and K_v_ to model the stability of the resting membrane potential. Figure 6A illustrates the regressions for the conductances for both ion channels. The resultant time series for vehicle (Figure 6B), oxaliplatin (Figure 6C), and paclitaxel (Figure 6D) on predicted membrane potentials suggests that oxaliplatin or paclitaxel treated neurons were more unstable than the control neurons. The estimated variance in the membrane potential for vehicle, oxaliplatin and paclitaxel treated neurons was 43.41, 296.66, and 286.25, respectively. These models suggest that the chemotherapy treated TRPM8 expressing neurons were more likely to fire action potentials spontaneously. This concept was further supported by the lower threshold potentials for the Na_v_ currents from the oxaliplatin and paclitaxel treated mice when compared to vehicle treated mice (Figures 5A,B).

**Figure 7:**
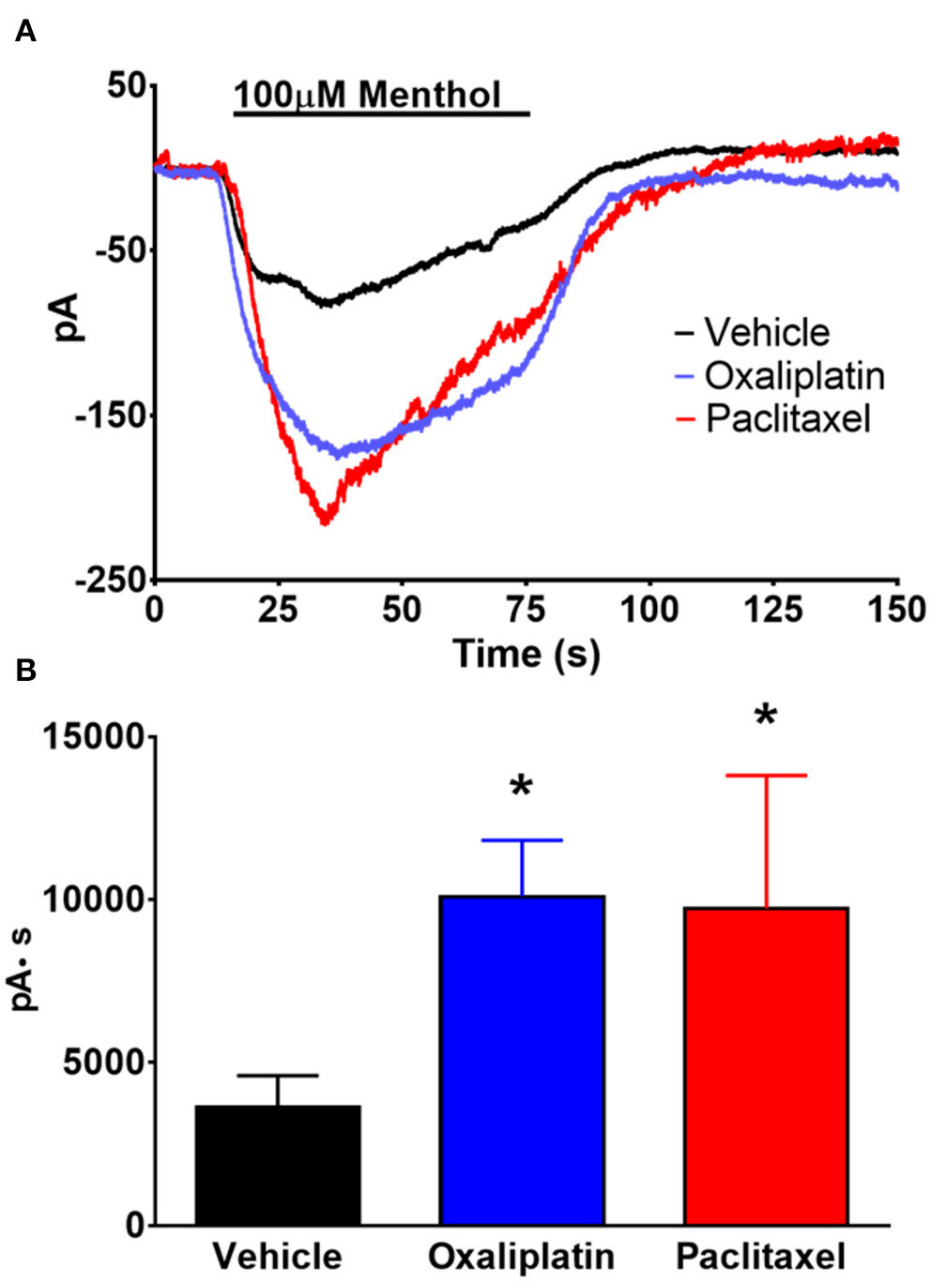
Effect of oxaliplatin and paclitaxel on menthol evoked currents in TRPM8 expressing TRG neurons. TRPM8^EGFP−/+^ mice were treated with oxaliplatin, paclitaxel, or PBS ip. as described in the methods. The TRG neurons were whole cell voltage clamped as described previously. **(A)** Bath application of menthol (100 μM) induced inward currents that were enhanced by prior treatment of the mice with either oxaliplatin or paclitaxel. The traces are averages of *N* = 27 neurons from 12 vehicle treated mice, *N* = 29 neurons from 8 oxaliplatin treated mice, and *N* = 15 neurons from 4 paclitaxel treated mice. **(B)** Area under the curve analysis of the currents in **(A)**. [ANOVA *F*_(2, 68)_ = 3.482, *P* = 0.0363]. Asterisks indicate *P* < 0.05 Dunnett's test when compared to vehicle.

Previous work by others demonstrated that TRPM8 mediated responses were enhanced by oxaliplatin and that there is an increase in the expression of TRPM8 in DRG neurons (5, 9). Since our previous study found that TRPM8 was not activated by cooling these neurons (12) we activated TRPM8 mediated currents in TRG neurons by superfusing the cells with the TRPM8 agonist menthol (100 μM). As demonstrated in Figures 7A,B prior treatment of the mice with either oxaliplatin or paclitaxel increased the amplitude of menthol induced currents in the TRPM8 expressing TRG neurons [ANOVA *F*_(2, 68)_ = 3.482, *P* = 0.0363, *N* = 27 neurons from 12 vehicle treated mice, *N* = 29 neurons from 8 oxaliplatin treated mice, *N* = 15 neurons from 4 paclitaxel treated mice].

### Immunohistochemistry

**Figure 8:**
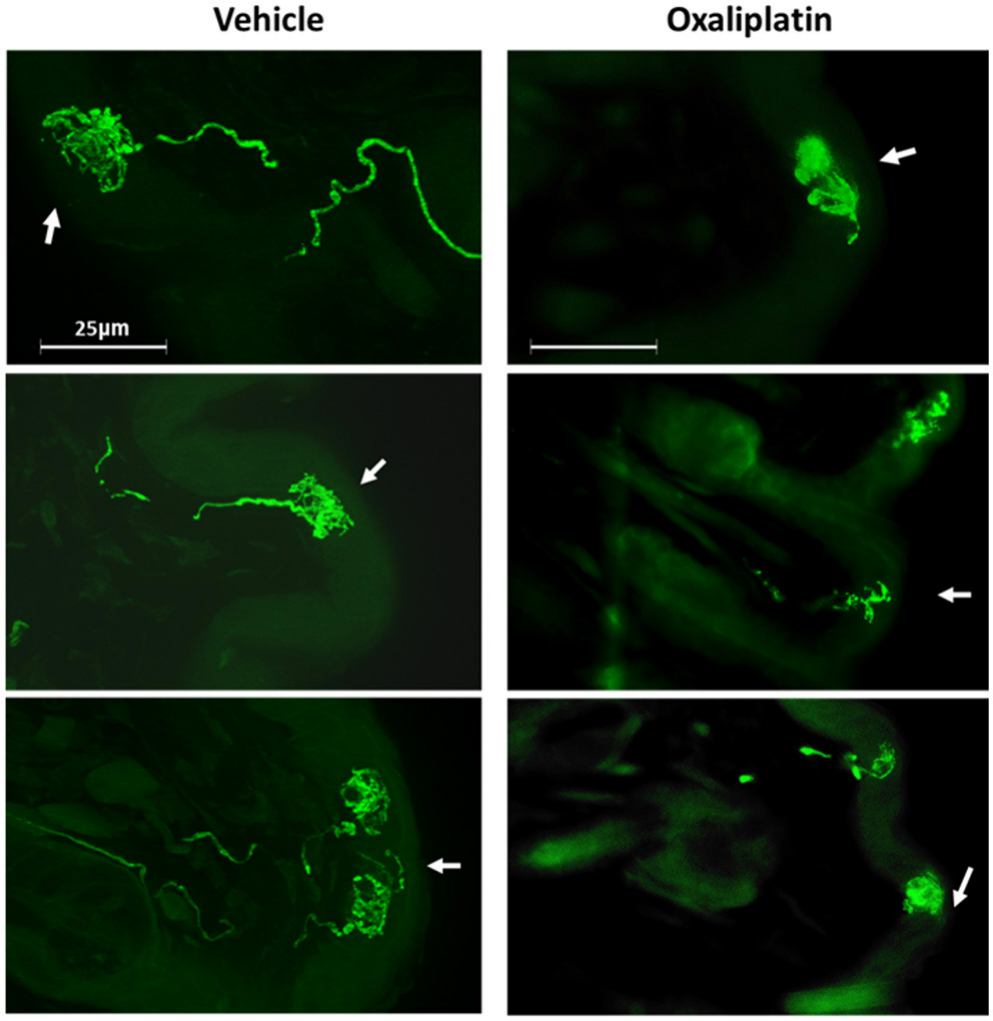
Effect of oxaliplatin on TRPM8 expressing TRG axons. TRPM8^EGFP−/+^ mice were treated with ip. oxaliplatin, or PBS. Six weeks following the last injection the mice were euthanized and the whisker pads were harvested, sectioned, and labeled for eGFP. The left column is three representative sections from vehicle treated mice and the right column is three representative sections from oxaliplatin treated mice. The arrows indicate the location of the surface of the skin.

The incongruence of the behavior data with the electrophysiology data in oxaliplatin treated mice suggested that the TRPM8 expressing TRG neurons may have lost communication with the skin, which prevented the cool temperatures from activating the sensitized neurons. To address this issue, we sectioned whisker pads of TRPM8^EGFP−/+^ mice that had been treated with ip. PBS or oxaliplatin 6 weeks prior to collection of the tissue. The sections were prepared as described in the methods section and examined under fluorescence microscopy. Axons from eGFP labeled neurons were identified in the whisker pads of PBS treated mice as illustrated in Figure 8. Many axons were observed to terminate near the surface of the skin in basket like structures. Work by Bouvier et al. demonstrated that these TRPM8 baskets wrap around Merkel cells and that Merkel cells respond to reduced temperatures (48). Dhaka et al., however, described these structures as bush/cluster free nerve endings (49). Following treatment with oxaliplatin the eGFP labeled axons were difficult to find and the basket structures were diffuse, smaller, and appeared damaged when compared to the vehicle treated baskets (Figure 8). The area of the baskets was quantified using ImageJ. The basket structures of vehicle treated animals had a mean area of 2,967.9 ± 592.8 μm^2^ and oxaliplatin treated animals had a mean area of 1,146.8 ± 167.2 μm^2^ (*t*-test, *t* = 2.740 df = 22, *P* = 0.012, *N* = 13 structures from 2 vehicle treated mice, *N* = 11 structures from 2 oxaliplatin treated mice). These findings indicated that the peripheral axons of TRPM8 expressing TRG neurons were damaged by oxaliplatin, likely within a few hours of the first treatment, resulting in a deafferentation of the neurons.

## Discussion

Oxaliplatin is commonly used to treat neoplasms such as colon cancer (50). An interesting side effect following treatment with oxaliplatin is an acute sensitivity to cool temperatures in the mouth and face (10, 11). The patients are generally warned to avoid cool or cold drinks and food to avoid this complication. These acute symptoms usually resolve and are not of significant concern in the long run. However, a longer-term neuropathy that includes numbness in the hands and feet and spontaneous pain may develop over the course of oxaliplatin therapy (1, 2). Our goal was to examine cool sensitivity in the face of mice following oxaliplatin treatment using our OPAD assay to determine the role of TRPM8 expressing TRG neurons in producing oxaliplatin's facial hypersensitivity. Our novel finding was that oxaliplatin damaged TRPM8 expressing axons in the face and that damage most likely disconnected these TRG neurons from the periphery.

In this study oxaliplatin treatment did not induce a detectable acute hypersensitivity to 18°C. We followed the animals in the OPADs for several more weeks to see if a prolonged hypersensitivity developed. Again, oxaliplatin did not produce the expected hypersensitivity (Figure 1B). We also treated mice with paclitaxel to determine if the OPAD assay could identify sensitivity to this chemotherapeutic agent. Although paclitaxel did not produce hypersensitivity in the OPAD assay to 18°C (Figure 1C) it did produce a long-lasting sensitivity to 42°C. Oxaliplatin treated mice, however, did not demonstrate facial hypersensitivity to the warmer temperature (Supplementary Figure 1). The data from the paclitaxel animals indicated that the OPAD assay could detect chemotherapy induced neuropathy in TRG innervated tissue. This finding was consistent with our previous work with paclitaxel (18). Our findings with oxaliplatin in the OPAD assay were surprising and inconsistent with published work demonstrating hypersensitivity in other types of behavioral assays (4–11, 34–37, 51–63).

Due to the reported cool sensitivity following oxaliplatin we expected that TRPM8 expressing trigeminal primary afferent neurons would be affected by the treatment. TRPM8 in primary afferent neurons was reported to be responsible for temperature detection in the range of ~15–22°C (13). However, we previously found that in mouse TRPM8 expressing TRG neurons the response to cool temperatures was not mediated by TRPM8, but rather by the differential effect of temperature on HCN and K_v_ channels (12). Orio et al. also reported that HCN channels play a role in cold sensitivity in trigeminal neurons (64, 65). Because of our previous work and the role of TRPM8 in temperature detection, we examined HCN, K_v_, Na_v_, and menthol activated TRPM8 currents to determine if oxaliplatin influenced TRPM8 expressing TRG neurons despite the lack of an observable behavioral effect in the OPAD assay. We acutely dissociated TRG neurons from vehicle, oxaliplatin, and paclitaxel treated TRPM8^EGFP−/+^ mice and whole cell voltage clamped eGFP labeled neurons. These experiments demonstrated that HCN (Figure 4A), Na_v_ (Figure 5A), and menthol (Figures 7A,B) evoked currents were enhanced when the mice had been treated with either oxaliplatin or paclitaxel 6 to 10 weeks prior to isolating the TRPM8 neurons. These findings are consistent with previous studies in DRG neurons (5, 8–10, 51). K_v_ currents were not influenced by the treatments (Figure 3). This finding contrasts with the decrease in K_v_ currents reported by Gu and colleagues (11, 66, 67); however, they examined the neurons at early time points following oxaliplatin treatment and they did not restrict their study to TRPM8 expressing neurons. Work by Ta et.al. demonstrated that TRPM8 transcripts were increased in DRG, but not in TRG 3 weeks following a regimen of oxaliplatin (37). Our increase in menthol evoked currents contradicts their findings but is consistent with the increase in TRPM8 reported in TRG by Gauchan et al. and Descoeur et al. (5, 8).

Modeling the membrane properties suggests that the observed changes in HCN produced by oxaliplatin and paclitaxel would decrease membrane stability and lead to an enhanced probability of the neurons firing action potentials (Figure 6). The variance in the predicted membrane potentials in the oxaliplatin and paclitaxel models increased over 6-fold as a result of the change in HCN currents. Furthermore, the Na_v_ current data not only indicated that the currents were increased by both chemotherapeutic agents, but the threshold potentials for the currents were shifted to more negative potentials (Figures 5A,B). These findings indicate that both the chemotherapy agents produced an enhanced probability of chronic spontaneous activity in the TRPM8 expressing TRG neurons. The spontaneous firing could indicate that the ganglionic cell bodies are driving chronic pain without peripheral input.

The disagreement between the behavior and electrophysiology experiments was resolved by examining the TRPM8 labeled axon terminals in the skin of the whisker pads. The OPAD Peltier probes contact the mice on the whisker pads and cheeks. In vehicle treated TRPM8^EGFP−/+^ mice the axons were abundantly visible in the skin and were observed to terminate in basket like structures. In the oxaliplatin treated mice, however, the axons were less abundant. Furthermore, the basket structures were smaller and diffuse in appearance, indicating that they had been damaged by the oxaliplatin (Figure 8). These findings suggest that the TRPM8 expressing TRG neurons' axons were damaged by the oxaliplatin within a few hours of the first treatment and that the resultant deafferentation prevented the animals from detecting the cool temperatures in the OPAD. However, the neurons' cell bodies survived the treatment, albeit with altered ion channels that could produce spontaneous firing. Bouvier et al. previously reported that the basket like structures surrounded Merkel cells (48). Thus, the deafferentation of Merkel cells by oxaliplatin may be associated with the numbness reported by patients.

One potential caveat to the conclusion that the lack of oxaliplatin induced facial hypersensitivity was due to neuronal degeneration is that we used SKH1 hairless mice for the behavior and TRPM8^EGFP−/+^ mice for the electrophysiology and immunohistochemistry. The TRPM8^EGFP−/+^ mice are on a C57BL/6 background. The reason SKH1 mice were used for the behavior experiments was that the hairless phenotype allows direct contact of the skin with the thermal probes. The fur on the faces of C57BL/6 mice insulates the animals from the stimulus. Thus, the cheeks of C57BL/6 mice would have to be shaved frequently during these behavioral studies, which produces irritation on the skin and possible hypersensitivity (14). Our previous work comparing thermal sensitivity in the face of SKH1, and C57BL/6 mice found that their temperature response profiles were qualitatively similar, but that the C57BL/6 licked on the reward bottle less than SKH1 mice, even at neutral temperatures. This finding indicated the possibility of mechanical allodynia in C57BL/6 mice due to the shaving of their cheeks (14).

The TRPM8^EGFP−/+^ mice were used in the study because their TRPM8 expressing neurons are labeled with eGFP making it possible to identify this class of neuron in tissue sections and *in vitro* for electrophysiology. It is possible that the two strains of mice respond to oxaliplatin differently and that the SKH1 mice are resistant to oxaliplatin induced neuropathies. However, we previously demonstrated that paclitaxel treatment produces thermal hypersensitivity in SKH1 mice that lasts for several weeks (18). Additionally, many strains of mice and rats have been used to study oxaliplatin induced neuropathies and the effects of the agent are similar across the board. The distinguishing feature of SKH1 hairless mice is that they have a mutation in the gene *hairless* that causes the nude phenotype. Mutations in this gene produce alopecia universalis in humans (68) and there are no reports of these individuals being resistant to oxaliplatin induced neuropathies. Therefore, we believe strain differences in the response to oxaliplatin are unlikely. However, future studies examining oxaliplatin thermal sensitivity on the paws of SKH1 mice or breeding the TRPM8^tm1Apat^/J mutation into the SKH1 background could answer this question.

One issue that remains unaddressed is the nature of the acute orofacial cool sensitivity produced by oxaliplatin. Our data suggests that the damage to the TRPM8 axon terminals/baskets happens within a few hours of the first treatment. It is possible that lower doses of oxaliplatin may have extended the duration of the acute cool temperature hypersensitivity so it could have been detected within the time frame of our OPAD testing. However, Zhao et al. also used a 10 mg/kg dose and found cool hypersensitivity in the paws within 2 h of the injection that lasted throughout their 1-week experiment (10). Thus, our data suggests the TRG neurons may be more susceptible to axonal damage than the DRG neurons. Our findings also suggest that the short-lived acute phase of oxaliplatin's effects on temperature sensitivity in orofacial regions may be terminated by deafferentation rather than by recovery of the neurons. This idea needs to be explored more thoroughly.

## Conclusion

This study examined the role of TRPM8 expressing TRG neurons in oxaliplatin induced cool temperature allodynia. The data indicates that the treatment leads to long-term enhanced activity in the TRPM8 expressing TRG neurons through alterations in HCN and Na_v_ ion channels, but oxaliplatin induced damage to the axons of these neurons prevents them from transducing temperature sensations from the skin.

## Data Availability Statement

The original contributions presented in the study are included in the article/Supplementary Material, further inquiries can be directed to the corresponding author/s.

## Ethics Statement

The animal study was reviewed and approved by University of Florida Institutional Animal Care and Use Committee.

## Author Contributions

RC wrote the manuscript. JN reviewed the manuscript. All authors contributed to the conception and design of the experiments, contributed to the article, and approved the submitted version.

## Funding

Funding was provided by the University of Florida Pain Research and Intervention Center of Excellence and the University of Florida Cancer Center.

## Conflict of Interest

The authors declare that the research was conducted in the absence of any commercial or financial relationships that could be construed as a potential conflict of interest.

## Publisher's Note

All claims expressed in this article are solely those of the authors and do not necessarily represent those of their affiliated organizations, or those of the publisher, the editors and the reviewers. Any product that may be evaluated in this article, or claim that may be made by its manufacturer, is not guaranteed or endorsed by the publisher.

## Supplementary Material

Supplemental Figure 1 Effect of oxaliplatin and paclitaxel at 42°C in the OPAD assay. SKH1 mice were treated with vehicle, oxaliplatin, or paclitaxel at the times indicated by the arrows and tested in the OPAD at 42°C. **(A)** Oxaliplatin treatment [2-Way Repeated Measures ANOVA *F*_(1, 44)_ = 0.1215, *P* = 0.7290, *N* = 10 oxaliplatin, *N* = 36 vehicle]. **(B)** Paclitaxel treatment [2-Way Repeated Measures ANOVA *F*_(1, 34)_ = 10.45, *P* = 0.0027, *N* = 16 paclitaxel, *N* = 20 vehicle].
