# Supplementary figures and images for: Effects of Oxaliplatin on Facial Sensitivity to Cool Temperatures and TRPM8 Expressing Trigeminal Ganglion Neurons in Mice
Source: Front Pain Res (Lausanne). 2022 May 11;3:868547. doi: 10.3389/fpain.2022.868547 (PMC9130462; doi:10.3389/fpain.2022.868547)

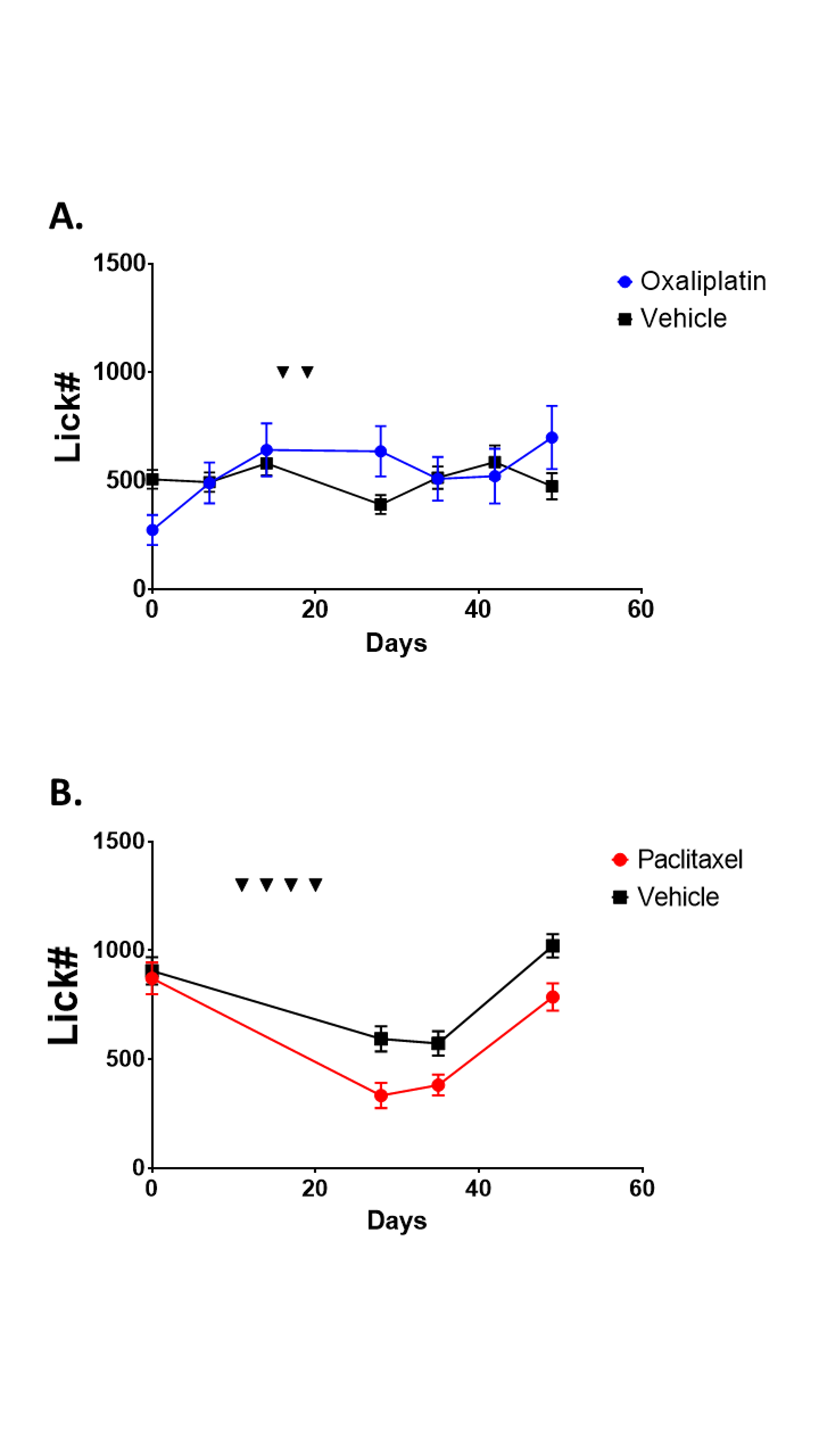

Supplement: Supplemental Figure 1 — Effect of oxaliplatin and paclitaxel at 42°C in the OPAD assay. SKH1 mice were treated with vehicle, oxaliplatin, or paclitaxel at the times indicated by the arrows and tested in the OPAD at 42°C. (A) Oxaliplatin treatment [2-Way Repeated Measures ANOVA F(1, 44) = 0.1215, P = 0.7290, N = 10 oxaliplatin, N = 36 vehicle]. (B) Paclitaxel treatment [2-Way Repeated Measures ANOVA F(1, 34) = 10.45, P = 0.0027, N = 16 paclitaxel, N = 20 vehicle]. [file Image_1.TIF]
